# Molecular and Behavioral Differentiation among Brazilian Populations of *Lutzomyia longipalpis* (Diptera: Psychodidae: Phlebotominae)

**DOI:** 10.1371/journal.pntd.0000365

**Published:** 2009-01-27

**Authors:** Alejandra S. Araki, Felipe M. Vigoder, Luiz G. S. R. Bauzer, Gabriel E. M. Ferreira, Nataly A. Souza, Izeneide B. Araújo, James G. C. Hamilton, Reginaldo P. Brazil, Alexandre A. Peixoto

**Affiliations:** 1 Laboratório de Biologia Molecular de Insetos, IOC, Fundação Oswaldo Cruz, Rio de Janeiro, Brazil; 2 Laboratório de Transmissores de Leishmanioses, IOC, Fundação Oswaldo Cruz, Rio de Janeiro, Brazil; 3 Curso de Ciências Biológicas e Agrárias, Universidade Estadual do Piauí, Parnaíba, Piauí, Brazil; 4 Centre for Applied Entomology and Parasitology, Institute of Science & Technology in Medicine, Keele University, Keele, Staffordshire, United Kingdom; 5 Laboratório de Bioquímica e Fisiologia de Insetos, IOC, Fundação Oswaldo Cruz, Rio de Janeiro, Brazil; Yale University, United States of America

## Abstract

**Background:**

*Lutzomyia longipalpis* is the primary vector of American visceral leishmaniasis. There is strong evidence that *L. longipalpis* is a species complex, but until recently the existence of sibling species among Brazilian populations was considered a controversial issue. In addition, there is still no consensus regarding the number of species occurring in this complex.

**Methodology/Principal Findings:**

Using *period*, a gene that controls circadian rhythms and affects interpulse interval periodicity of the male courtship songs in *Drosophila melanogaster* and close relatives, we analyzed the molecular polymorphism in a number of *L. longipalpis* samples from different regions in Brazil and compared the results with our previously published data using the same marker. We also studied the male copulation songs and pheromones from some of these populations. The results obtained so far suggest the existence of two main groups of populations in Brazil, one group representing a single species with males producing Burst-type copulation songs and cembrene-1 pheromones; and a second group that is more heterogeneous and probably represents a number of incipient species producing different combinations of Pulse-type songs and pheromones.

**Conclusions/Significance:**

Our results reveal a high level of complexity in the divergence and gene-flow among Brazilian populations of the *L. longipalpis* species complex. This raises important questions concerning the epidemiological consequences of this incipient speciation process.

## Author Summary

*Lutzomyia longipalpis* is the main vector of visceral leishmaniasis in the Americas. There is strong evidence that *L. longipalpis* is a species complex, but there is still no consensus regarding the number of species occurring in Brazil. We combined molecular and behavioral analyses of a number of *L. longipalpis* populations in order to help clarify this question. This approach has allowed us to identify two main groups of populations in Brazil. One group probably represents a single species distributed mainly throughout the coastal regions of North and Northeast Brazil and whose males produce the same type of copulation song and pheromone. The second group is more heterogeneous, probably represented by a number of incipient species with different levels of genetic divergence among the siblings that produce different combinations of copulation songs and pheromones. The high level of complexity observed raises important questions concerning the epidemiological consequences of this incipient speciation process.

## Introduction

Cryptic speciation is an interesting and important issue to evolutionary biologists as organisms that are distinct in several ways can look the same even to specialist taxonomists, leading to false conclusions about their biology. Moreover, it also has practical implications for conservation management and in the identification of economically or medically important species [1]. In addition, the study of cryptic speciation in blood-sucking insects can be epidemiologically relevant as sibling species might differ in their importance as disease vectors. One classical example is the *Anopheles gambiae* complex whose siblings differ in their host preference and other biological characteristics that together define very different vectorial capacities (reviewed in [2]).

*Lutzomyia longipalpis* (Lutz & Neiva 1912) is the primary vector of American visceral leishmaniasis in the Neotropics. This sand fly has an extensive and discontinuous distribution from southern Mexico to northern Argentina, and is found in a range of different habitats [3]–[5]. Geographic isolation between numerous populations of *L. longipalpis* favors the process of genetic divergence and cryptic speciation [6]–[12].

*L. longipalpis* males show a polymorphism in the number of abdominal spots, with either a single pair on the fourth tergite (1S) or two pairs, on the third and fourth abdominal segments (2S) [13]. Experimental evidence for a *L. longipalpis* complex was first obtained by crosses between Brazilian populations with different abdominal spot morphology [14],[15]. It was shown that the spot phenotype cannot be used as a reliable marker to identify different species because some crosses between populations with different male phenotypes did not show reproductive isolation, while some crosses between populations with the same male phenotypes had reduced insemination rates. However, in at least one specific locality (Sobral, Ceara State), males of the two forms found in sympatry represented different species that showed strong reproductive isolation [14],[15] suggesting that this morphological variation might still be a useful tool to distinguish between sympatric siblings in some situations.

After this early work many studies have been carried out on populations from different Latin American countries to determine the taxonomic status of this important Leishmania vector, indicating that *L. longipalpis* is a species complex. However there is no consensus on the delineation between members of the complex as different genetic markers suggest different conclusions (reviewed in [10]).

The fact that some genetic markers show clear evidence for a complex while others do not, strongly suggests recent or incipient speciation events that are perhaps also associated with introgression among the siblings. Therefore the data for the *L. longipalpis* populations in Brazil and in Latin America as a whole suggest a level of complexity that resembles what is found in the *Anopheles gambiae* complex and *An. gambiae s.s.* [2],[10]. In addition, it is still not clear how many species or incipient species of the *L. longipalpis* complex exist within Brazil. This is not only interesting from an evolutionary point of view but may also be epidemiologically relevant since most cases of visceral leishmaniasis in Latin America occur in the northeast region of Brazil. The characterization of the number, distribution and genetic divergence of the different species of the *L. longipalpis* complex might allow the future study of possible coevolutionary interactions between the different siblings and parasite genotypes that might influence *Leishmania* transmission, virulence and clinical outcome [16], as has been recently suggested for malaria [17].

Molecular markers associated with sexual behavior and reproductive isolation, such as the *period* (*per*) gene [18], are useful tools for speciation studies. In Drosophila, *per* is one of the genes that control circadian rhythms of emergence from pupae and locomotor activity in adult flies [19]. This clock gene also controls species-specific differences in the courtship song rhythms that are involved in reproductive isolation between *Drosophila melanogaster* and its sibling species [18], [20]–[22] and for that reason it was considered an example of “speciation gene” [23]. In addition *per* is usually highly variable and was widely used for population genetics studies in Drosophila [24].

The *per* gene was also shown to be a good molecular marker for studying the differentiation among populations of the *L. longipalpis* complex. Bauzer *et al.* [25] used the *per* gene to study three allopatric populations from Brazil (Natal, Lapinha and Jacobina) and showed that they were genetically highly differentiated. Later, using the same marker, Bauzer *et al.* [26] provided the first molecular evidence in Brazil (Sobral) for the existence of two genetically highly differentiated sympatric populations, confirming therefore the early results of Ward *et al.* [14],[15]. These findings were later corroborated by analysis of microsatellites [27],[28] and genetic differentiation in the *cacophony* (*cac*) gene [29].

Acoustic signals are an important component of courtship behavior in insects [30] and they show rapid evolution in some Drosophila sibling species (e.g. [31]). As in Drosophila, acoustic communication is important in *L. longipalpis* sexual behavior [32],[33]. However, unlike *D. melanogaster* [34], but similar to some other Drosophila species [35], males of *L. longipalpis* vibrate their wings during copulation. Two main types of copulatory courtship songs, named Pulse-type and Burst-type, were found in six Brazilian populations [32]. The Burst-type song (B), composed of trains of extremely polycyclic pulses (“bursts”) modulated in frequency and amplitude, showed no significant variation among the three populations producing this pattern (Natal, Sobral 2S and Marajó) [32]. On the other hand the Pulse-type song is more variable and three different patterns (P1, P2 and P3) were identified in the populations of Jacobina, Lapinha and Sobral 1S, respectively [32]. The P1 songs are composed of trains of pulses with usually two or three cycles per pulse. P2 songs differ from P1 mainly by the presence of interspersed polycyclic pulses between nearly monocyclic pulses. The P3 songs are characterized by an almost perfect alternation of high and low amplitude pulses [32]. These data suggest the existence of four different siblings among these six populations [10],[16].

Males of the *L. longipalpis* complex also produce different types of terpenoid volatile compounds with either 16 or 20 carbons that function as sex pheromones [36]–[41]. Interestingly, the Pulse-type populations mentioned above produce different types of C16 pheromones while Burst-type populations produce the same type of C20 [32]. The two groups also show reproductive isolation in crossing experiments [14],[15],[42] and a clear divergence with microsatellites, *cac* and *per* gene sequences [25]–[29],[32].

Integrative approaches are particularly useful to study cryptic speciation as not all characters are necessarily going to show the same level of differentiation and studying multiple, different types of characters will more fully describe any differentiation [1]. In the present study, we carried out a combined analysis of *per* gene molecular polymorphisms, copulation songs and pheromones from a number of Brazilian *L. longipalpis* populations comparing new data we obtained to previously published results. One of our underlying hypotheses was that more species of the complex must exist in Brazil since previous molecular and behavioral analysis of the six populations described above revealed the existence of four siblings. A second hypothesis was that other sympatric siblings would probably be found in localities where *L. longipalpis* males with one and two spots coexist as observed in Sobral. Finally, because copulation songs and pheromones are likely to evolve under sexual selection, our third underlying hypothesis was that the observed correlation between genetic divergence in molecular markers and these two traits would not necessarily hold once a large number of populations was analyzed.

## Methods

### *period* gene analysis

**Figure 1:**
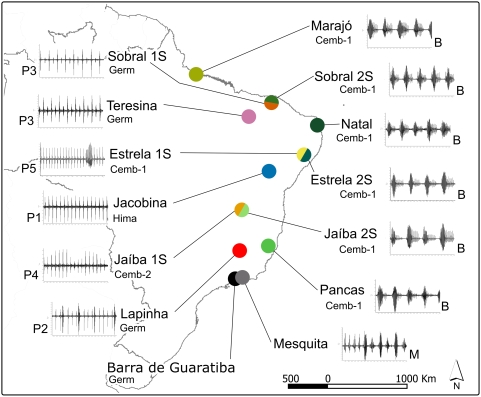
Sample sites and summary of the information available for the different copulation songs and main pheromone types from 14 different *L. longipalpis* populations from Brazil. Copulation songs: Burst-type (B) and Pulse-types (P1, P2, P3, P4, and P5); Pheromone types: cembrene (Cemb), 9-methylgermacrene-B (Germ) and himachalene (Hima). Cemb-1 and Cemb-2 differ in the mass spectra shape and ion composition of the most abundant diterpene compounds [53]. The copulations songs of Lapinha, Jacobina, Marajó, Natal and Sobral were previously described in [32],[33] while the pheromone data for the populations of Lapinha, Jacobina, Jaíba, Natal, Marajó and Sobral were published in [33], [36]–[38],[53].

We analyzed *Lutzomyia longipalpis* samples from eight populations (see below) collected in six Brazilian localities: Barra de Guaratiba (23°04′S, 43°35′W), Rio de Janeiro State; Estrela de Alagoas (09°23′S, 36°45′W), Alagoas State; Jaíba (15°20′S, 43°41′W), Minas Gerais State; Mesquita (22°46′S, 43°25′W), Rio de Janeiro State; Pancas (19°13′S, 40°51′W), Espírito Santo State; and Teresina (05°05′S, 42°48′W), Piauí State. A map with the approximate position of these localities is shown in Figure 1. Species identification was carried out according to Young and Duncan [3]. In most cases the specimens analyzed were either wild-caught males or the F1 males of different wild-caught females. However, in the case of Mesquita, due to the difficulties explained below, the sequences were obtained from the F1 of a single wild-caught female. In the samples of Estrela de Alagoas and Jaíba, where males present either one (1S) or two spots (2S), the samples of the two morphotypes were considered potential sympatric siblings and therefore were analyzed separately. In Teresina nearly 95% of the flies were 1S and only those were used in the analysis. The remaining analyzed samples were also all 1S.

Genomic DNA extraction was carried out as in Jowett [43]. The PCR was done as described in Bauzer *et al.* [25]. The PCR fragments were purified using the Wizard PCR Prep kit (Promega) and cloned using the pMOS Blunt Ended cloning kit (GE Healthcare) or the pGEM T Easy Vector System I (Promega). The plasmid DNA was prepared with the Flexiprep kit (GE Healthcare) or using alkaline lyses method in micro-plates of 96 wells [44] and filtered in Multiscreen-filter plates. Sequencing of cloned fragments was carried out using the ABI Prism Big Dye Terminator Cycle Sequencing Ready Reaction V3.0 or V3.1 kits (Applied Biosystems) in ABI Prism 377 and ABI 3730 DNA Sequencers at Fundação Oswaldo Cruz, Rio de Janeiro, Brazil.

From three to eight sequences were obtained for each individual corresponding to the eight populations. In most cases the sequences were aligned generating a single consensus sequence per individual. For individuals with 6 or more sequences available, two consensus sequences representing both alleles were generated (denominated haplotypes A and B). Analysis of the data including and excluding these extra sequences showed no significant differences and therefore they were also used. The sequences have been submitted to GenBank (accession numbers EU713077 to EU713233).

The DNA sequences were edited and aligned using the Wisconsin Package Version 9.1; Genetic Computer Group (GCG) (Madison, Wisconsin, USA). The polymorphism analyses and three neutrality tests were performed using DnaSP 3.5 [45] and differentiation between populations was estimated using ProSeq program [46]. The genealogy of the sequences was carried out using MEGA 3.1 [47]. The analysis of molecular variance (AMOVA) was carried out using Arlequin 3.1 [48]. For these analyses the 113 sequences obtained by Bauzer *et al.* [25],[26] from the populations of Jacobina, Lapinha, Natal and Sobral (1S and 2S), and the five sequences from Salvaterra (Marajó Island, Pará State) from Souza *et al.* [32] were considered as well.

The haplotype networks were constructed using either the whole fragment of 266-bp or a 58-bp non-recombinant block selected after recombination analysis [49] available in DnaSP 3.5 and exclusion of a few recombinant sequences. The networks were estimated using statistical parsimony [50] implemented in the TCS1.21 software [51].

### Copulation song analysis

We recorded and analyzed male copulation songs from seven samples: Estrela de Alagoas (one and two spot males); Jaíba (one and two spot males), Pancas, Teresina and Mesquita. We attempted to obtain new samples of live males from Barra de Guaratiba, to record their songs but this population was found to be very small (see below) and ephemeral and consequently we were not successful. *L. longipalpis* is very rare in Mesquita, which is dominated by *L. intermedia* (Nataly A. Souza, unpublished results) and the collection area is surrounded by a “Favela” (shanty town) that is currently inaccessible to new collections. Therefore only the progeny of a single female was analyzed and no material was available for pheromone analysis (see below). In addition, we analyzed the songs of four different Mesquita copulations but three of the four songs were produced by the same male copulating with three different females. The three songs produced by this male were included in the analysis because the variation in the song parameters among these three copulations was higher than the variation observed between the songs of the two different males (data not shown).

Songs were recorded as in Souza *et al.* [32]. A male and a female were placed in a small mating chamber inside the INSECTAVOX [52] for about five minutes. If there was no copulation after 5 minutes the couple was replaced. Usually only one copulation is observed in every 15 to 20 trials. The recordings were carried out at 26°C±1°C using a Sony Hi8 CCD-TRV65 videocamera and a Sony SLV-77HFBR VCR. Some individuals from Jaíba, both 1S and 2S, and from Estrela de Alagoas 1S were recorded using a DVD recorder, Panasonic DMR-ES10. Most recordings were carried out using F1 virgin individuals (between 3 to 8 days old) reared in the lab from wild-caught females. A number of the males that had their songs recorded were also used in the molecular analysis.

The songs were digitalized on a CED 1401 A/D converter and analyzed using Spike 2 software (version 4.08), both from Cambridge Electronic Design (UK). The song parameters analyzed were: interpulse (IPI) or interburst interval (IBI), number of pulses or bursts per train (NT), train length (TL), cycles per pulse (CPP), and carrier frequency of train (Freq). In the Jaíba 1S and Teresina populations most parameters were similar (see below) but the songs of the latter tended to have pulses that alternated in amplitude, similar to what was previously seen in Sobral 1S [32]. Therefore, for these two populations we performed an analysis of the pulse amplitude alternation (AmpAlt) looking at the proportion of pulses that have amplitudes either lower or higher than the flanking pulses. The statistical analysis was carried out using STATISTICA (version 5, StatSoft, Inc., USA).

### Pheromone analysis

Individual males were placed in flame-cleaned Pasteur pipette ampoules and sufficient pesticide-grade hexane (∼10 ul) (BDH, Poole, Dorset, U.K) was added to cover them and extract the sex pheromone. The ampoules were heat-sealed and stored at −20°C until analysis [38].

Hexane extracts containing the sex pheromones were analyzed by Coupled Gas Chromatography - Mass Spectrometry on a HP-5MS capillary column, 30 m×0.25 mm i.d., 0.25 µm film thickness (Agilent, Stockport, Cheshire) in a Hewlett Packard 5890 II+ Gas Chromatograph coupled to a Hewlett Packard 5972A bench top mass spectrometer (electron impact, 70 eV, 180°C). Injection of the sample was via a heated injector (170°C). The carrier gas was helium at 1 ml/min. The GC was temperature-programmed with an initial 2 min at 40°C, then a rise of 10°C/min to a final isothermal period at 250°C (4 min).

We analyzed the pheromones of samples from Barra de Guaratiba, Teresina and Pancas. We were not able to obtain a new sample from Mesquita, due to the reasons stated above. Information on the pheromones of the other populations included in this work is already published [33], [36]–[38],[53].

## Results

### Copulation song and pheromone analyses

Our previous work [32] has shown that the copulatory courtship songs produced by *L. longipalpis* males have two main components: a primary song produced by all males during copulation that shows remarkable differences among some populations, and a secondary song that is composed of low amplitude polycyclic pulses with highly variable intervals. The secondary song is not produced by every male and when it occurs it is usually seen between two primary song trains [32]. In the present study we also observed that the low amplitude secondary song seems to be similar in all populations and it was not analyzed in detail. However, analysis of the primary song indicates striking differences among the studied samples (song traces are shown in Supplemental Figure S1).

Males from Pancas and two spot males (2S) from Estrela de Alagoas and Jaíba produce the same type of Burst song previously observed in Natal, Marajó and Sobral 2S males [32] (see Figure 1 and Figure S1). In the sample from Teresina we observed the same Pulse-type song (P3) previously recorded in the population of Sobral 1S [32] (Figure 1). In addition, two new types of Pulse songs were observed in Jaíba 1S and Estrela 1S males that were called P4 and P5, respectively (Figure 1 and Figure S1) (see below for a description of these new Pulse songs). Note that 1S and 2S males from Jaíba and Estrela produce very distinct songs indicating that in both localities the spot phenotype might be associated with different sympatric species as observed in Sobral, and that is consistent with the molecular data (see below). Finally, males from Mesquita produce a new song type that seems to present a mixed (M) pattern between Burst and Pulse songs (Figure 1 and Figure S1).

Table 1 shows the mean (±SEM) for the different song parameters. Because of the very different patterns observed in Burst and Pulse-type songs their statistical analyses were carried out separately comparing the data to our previously published results for other populations [32].

**Table 1:** Copulation song parameters of the analyzed populations.

	N	Type	IPI / IBI (ms)	NP	TL (s)	Freq (Hz)	CPP	AmpAlt
**Estrela 1S**	5	P	36.59±0.89	101.30±4.23	3.66±0.08	174.05±0.43	1.99±0.16*	-
							7.58±0.77**	
**Jaíba 1S**	4	P	66.68±4.12	33.00±6.81	2.10±0.36	298.39±7.67	2.81±0.33	0.17±0.13
**Teresina**	7	P	65.50±0.74	32.81±2.75	2.08±0.18	298.67±9.05	3.11±0.15	0.86±0.04
**Estrela 2S**	9	B	234.97±14.01	13.07±0.95	2.97±0.26	275.88±8.05	-	-
**Jaíba 2S**	8	B	279.12±14.36	10.78±1.07	2.89±0.34	272.11±11.60	-	-
**Pancas**	5	B	224.56±11.92	12.00±1.00	2.58±0.14	291.50±4.20	-	-
**Mesquita**	4	M	106.91±3.40+	40.00±1.96+	4.14±0.29+	317.39±8.46+	13.94±0.20+	-
			89.85±6.91++	10.50±0.96++	0.86±0.08++	205.08±36.97++	7.98±0.39++	
			116.32±2.01+++	29.25±2.59+++	3.29±0.29+++	312.5±10.55+++	16.13±0.41+++	

*: value excluding polycyclic pulses.

**: value referring to the polycyclic pulses.

**+:** Values for the whole train.

**++:** Values of the pulse-like train segment.

**+++:** Values of the burst-like train segment.

P: pulse-type; B: burst-type; M: mix-type song.

Analysis of variance (ANOVA) comparing the Burst-type songs of the populations of Pancas, Estrela 2S, Jaíba 2S (this study), Natal, Marajó and Sobral 2S [32] detected a significant difference only for IBI (F_[5,38]_ = 3.89; p<0.01). We performed a Post-Hoc LSD (“Least Significant Difference”) test for this parameter and found that the difference occurs mainly because of the high IBI found in the population of Jaíba 2S (data not shown).

An ANOVA was also carried out comparing the populations that produce Pulse-type songs: Teresina, Jaíba 1S and Estrela de Alagoas 1S (this study), Jacobina, Lapinha and Sobral 1S [32]. The results showed significant differences in all parameters analyzed (IPI: F_[5,45]_ = 43.76; NP: F_[5,45]_ = 49.18; TL: F_[5,45]_ = 12.97; Freq: F_[5,45]_ = 25.18; CPP: F_[5,45]_ = 23.09; AmpAlt: F_[2,17]_ = 56.42; p<0.0001 in all cases).

As mentioned above males from Teresina sing like Sobral 1S males. They produce a P3 type song which shows an alternation of high and low amplitude pulses along the train with a ∼65 ms mean inter-pulse interval and about 2 s train length (Table 1). Post-Hoc LSD tests show no significant differences between Teresina and Sobral 1S males in any of the parameters analyzed (data not shown).

The song produced by Jaíba 1S males, named P4 (Figure 1 and Figure S1), has similar mean parameter values to the songs of Teresina and Sobral 1S (Table 1; [32]). However, the pattern is different and the P4 song does not show the alternating high and low amplitude pulses observed in the P3 song (Figure 1 and Figure S1). In P4 songs, the pulse amplitude grows and decreases gradually through out the train. Post-Hoc LSD analysis between Jaíba 1S and the populations of Sobral 1S and Teresina showed a significant difference only in the proportion of alternated amplitude pulses (AmpAlt) (data not shown).

Estrela de Alagoas 1S males produce the type 5 Pulse song (P5). This pattern presents similar pulses along the train with a few polycyclic pulses in the end (Figure 1 and Figure S1). This song has a very short IPI (∼37 ms) and a long train (∼3.6 s) compared with the other populations of *L. longipalpis* (Table 1) [32]. Post-Hoc LSD analysis shows significant differences in all parameters in most comparisons (data not shown).

Mesquita males produce a new song pattern that we called Mix (M). The M song type is characterized by the presence of polycyclic pulses in the first third of the train followed by highly polycyclic pulses resembling bursts at the end (Figure 1 and Figure S1). Table 1 shows the means (±SEM) of analyzed parameters for the whole song and for the more Pulse-like and Burst-like segments. These values show a clear separation of the two parts that constitute this new song pattern. ANOVA and LSD analysis were carried out comparing the two segments, the first segment to the populations with Pulse song and the second segment to the populations with Burst song. Significant differences were obtained in most comparisons (Table S1).

Finally, Table 2 shows the results of the Gas Chromatography and Mass Spectrometry analysis. In Pancas, males produce only diterpenes (20 carbon terpenes with a molecular weight of 272 amu) which can be characterized as cembrenes. The main compound found in males from this locality (R_t_ = 22.95) has a mass spectrum similar to the main cembrene-1 present in Sobral 2S males and some other populations [38]. The main compound found in the male pheromones of Barra de Guaratiba and Teresina is (*S*)-9-methylgermacrene-B (9MGB), a 16 carbon terpene with a molecular weight of 218 amu. However, males from these two localities, particularly Barra de Guaratiba, also produce several diterpenes.

**Table 2:** Gas chromatographic relative time (R*t*) in minutes, quantities (ng) and percentage composition (in brackets) of the terpene components found in hexane extracts from male *L. longipalpis* collected from 3 locations in Brazil; Barra de Guaratiba (BG), Pancas and Teresina.

Pheromone type	R*t* (min)	BG (n = 6)	Pancas (n = 13)	Teresina (n = 14)
9MGB	14.96	10.85^a^±2.65 (54.4%)	x	8.76^a^±3.98 (88.1%)
	21.01	2.08±0.68 (10.4%)	x	x
	22.28	2.20^b^±0.60 (11.0%)	x	0.87^b^±0.22 (8.8%)
diterpenes (cembrene isomers)	22.79	x	x	x
	22.95	3.06^c^±0.85 (15.3%)	2.36^c^±0.43 (89.4%)	x
	23.08	1.77^d^±0.54 (8.9%)	0.28^d^±0.16 (10.6%)	x
	21.12	x	x	0.31±0.06 (3.1%)

Peaks with superscript *a* are confirmed as (*S*)-9-methylgermacrene-B (9MGB) (mw 218 amu). Other peaks are characterized as diterpenes (cembrene isomers) (mw 272 amu) and those with the same superscript *b*, *c*, or *d* have mass spectra which match each other closely; x indicates that the compound was not found in the extracts; n is the number of individuals of each population examined.

Figure 1 shows a map with the approximate geographic position of the different localities with a summary of the information available for the different copulation songs and main pheromone types from 14 different populations based on this study and previously published data [32], [33], [36]–[38],[53]. This information will be compared to the results of the *period* gene molecular analysis that follows.

### *period* gene polymorphism and differentiation analysis

We amplified the same 266-bp fragment of the *L. longipalpis per* gene used by Bauzer *et al.* [25],[26] which includes a 54-bp intron. This fragment encodes part of the region between the PAS dimerization domain and the *Thr*-*Gly* region, including most of the cytoplasmic localization domain (CLD). We analyzed 157 sequences of the *L. longipalpis per* gene obtained from six localities (Barra de Guaratiba, Estrela de Alagoas, Jaíba, Mesquita, Pancas and Teresina). The sequences from Estrela de Alagoas and Jaíba were separated according to the male spot phenotypes (1S and 2S). The sequences from Marajó were included in this analysis as they were not analyzed in detail in Souza *et al.* [32]. For the 266 sites analyzed, 56 (21.05%) were variable. Most of the base substitutions were silent or occurred in the intron (see alignment in Figure S2). We found five non-silent substitutions occurring in four different sequences from Estrela de Alagoas 1S, all in the second exon. We also observed deletions which included either one or two sites in the intron region of two sequences from Estrela de Alagoas 2S and 18 sequences from Estrela de Alagoas 1S. The remaining sequences did not show any deletions or amino acid changes (Figure S2).

The number of segregating sites (S), nucleotide diversity (π) and the neutral parameter (θ) were calculated for each population (Table 3). Interestingly, the Barra de Guaratiba population did not present polymorphism in any of the 24 sequences analyzed. The samples from Estrela de Alagoas 1S and Jaíba 1S were the most polymorphic of the *L. longipalpis* samples analyzed. We carried out three tests of selective neutrality, Tajima's D [54], Fu's F_S_ [55] and Ramos-Onsins and Rozas' R_2_ [56]. In all cases the values were not significant after Bonferroni's correction (Table 3), indicating no obvious departures from neutrality in *L. longipalpis per* gene.

**Table 3:** Polymorphism summaries.

Sample	n_i_	n_s_	S	π		θ		D_T_	F_S_	R_2_
**Barra de Guaratiba**	24	24	0	0	-	0	-	-	-	-
**Estrela 1S**	15	22	33	0.0225	(0.000025)	0.0343	(0.000158)	−1.4748	−2.0760	0.0888
**Estrela 2S**	21	30	30	0.0187	(0.000009)	0.0286	(0.000101)	−1.3976	−6.7757	0.0724
**Jaíba 1S**	10	17	20	0.0221	(0.000003)	0.0222	(0.000082)	−0.2059	−7.7875	0.1216
**Jaíba 2S**	10	15	18	0.0178	(0.000005)	0.0208	(0.000077)	−0.5816	−3.5445	0.1058
**Marajó**	4	5	12	0.0196	(0.000044)	0.0217	(0.000145)	−0.7031	2.3849	0.2102
Mesquita*	3	4	4	0.0088	(0.000008)	0.0082	(0.000031)	-	-	-
**Pancas**	24	27	20	0.0134	(0.000003)	0.0195	(0.000054)	−0.5378	−9.2388	0.0792
**Teresina**	17	18	14	0.0137	(0.000004)	0.0153	(0.000043)	−0.4034	−4.8773	0.1177

n_i_, number of individuals and n_s_, number of DNA sequences of the samples (see Methods for further details); S number of polymorphic sites; π, average number of pair-wise differences; θ, neutral parameter, based on the number of segregating sites. Variances of π and θ values are shown in brackets. D_T_, Tajima's D [54]; F_S_, Fu's F_S_ [55] and R_2_, Ramos-Onsins and Rozas' R_2_ [56]. ^*^The Mesquita sequences were derived from the F1 of a single female and therefore no tests were carried out with this sample.

For the analysis of molecular differentiation all pair-wise comparisons were performed including the five populations obtained by Bauzer *et al.* [25]–[26] plus the samples of *L. longipalpis* analyzed in this study, excluding the monomorphic Barra de Guaratiba population and the Mesquita sample that was derived from the F1 of a single female. Table 4 shows the pair-wise fixation index (F_ST_) [57], and Tables S2 and S3 show the number of shared/fixed polymorphic sites and the number of exclusive polymorphic sites, respectively. The results of the F_ST_ analysis clearly indicate that the 1S and 2S males from Estrela de Alagoas (AL) and Jaíba (MG) belong to different sympatric species. Therefore we started analyzing in more detail these two localities before carrying out a general analysis of all populations.

**Table 4:** Population subdivision statistics F_ST_ for Brazilian populations of *L. longipalpis*.

		[--------------- Pulse-type populations ------------------]					[------------------ Burst-type populations -------------------]					
		J1S	Ter	Jac	Lap	S1S	E2S	J2S	Mar	Pan	Nat	S2S
**Pulse-type populations**	**E1S**	0.339^***^	0.378^**^	0.400^***^	0.403^***^	0.271^***^	0.428^***^	0.489^**^	0.539^ns^	0.529^***^	0.509^***^	0.495^***^
	**J1S**		0.098^*^	0.367^***^	0.043^ns^	0.049^ns^	0.315^***^	0.372^**^	0.415^ns^	0.407^***^	0.396^**^	0.368^**^
	**Ter**			0.408^***^	0.175^**^	0.067^ns^	0.371^***^	0.434^**^	0.508^ns^	0.491^***^	0.463^***^	0.441^***^
	**Jac**				0.404^***^	0.211^***^	0.452^***^	0.507^**^	0.555^ns^	0.548^***^	0.524^**^	0.514^***^
	**Lap**					0.124^***^	0.386^***^	0.437^**^	0.484^ns^	0.471^***^	0.466^***^	0.434^***^
	**S1S**						0.323^***^	0.388^**^	0.464^ns^	0.436^***^	0.406^***^	0.395^***^
**Burst-type populations**	**E2S**							0-^ns^	0.164^ns^	0.041^*^	0.003^ns^	0.011^ns^
	**J2S**								0.123^ns^	0.029^ns^	0-^ns^	0-^ns^
	**Mar**									0.142^ns^	0.180^ns^	0.116^ns^
	**Pan**										0.041^ns^	0.019^ns^
	**Nat**											0-^ns^

Significance of F_ST_ values was evaluated by 1000 random permutations (* = p<0.05, ** = p<0.01, *** = p<0.001, ns (non-significant) = p>0.05). E1S: Estrela 1S, J1S: Jaíba 1S, Ter: Teresina, Jac: Jacobina, Lap: Lapinha, S1S: Sobral 1S, E2S: Estrela 2S, J2S: Jaíba 2S, Mar: Marajó, Pan: Pancas, Nat: Natal, S2S: Sobral 2S). 0- Negative values.

### Analysis of putative sympatric siblings in Estrela de Alagoas and Jaíba

A highly significant genetic differentiation was found between Estrela 1S and 2S, a result that is similar to that observed between Sobral 1S and 2S [26]. Although no fixed differences in *per* were observed between Estrela 1S and 2S, perhaps due to introgression (see below), the number of exclusive and shared polymorphisms is similar (20 and 19, respectively) (Tables S2 and S3). In addition, the results presented in Table 4 suggest that Estrela 2S is essentially the same gene pool as Jaíba 2S, Natal, Marajó, Sobral 2S and Pancas, with all F_ST_ values low and/or non-significant. This is consistent with the song analysis presented above. On the other hand all comparisons involving samples from Estrela 1S showed high levels of differentiation. The lowest F_ST_ value for Estrela 1S was observed in the comparison with Sobral 1S.

**Figure 2:**
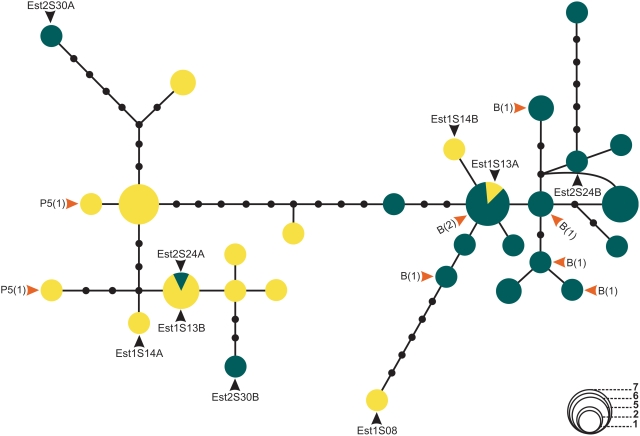
Haplotype network of the *L. longipalpis* sequences from Estrela de Alagoas. Estrela 1S (yellow) and Estrela 2S (green). Haplotypes from individuals with recorded copulation songs (B: Burst-type and P5: Pulse-type 5) and the number of sequences (in brackets) are shown. Curved lines represent alternative branching between haplotypes.

Figure 2 shows a haplotype network of all sequences from Estrela de Alagoas (1S and 2S) (see also Figure S3 for a minimum evolution tree of the same sequences). The haplotypes are clearly separated into two major groups. The first one consists mainly of Estrela 1S sequences, plus three haplotypes of Estrela 2S. The second group consists of Estrela 2S sequences, plus three haplotypes of Estrela 1S. Note in Figure 2 the few available sequences from Estrela males that have had also their songs analyzed. Haplotypes from males producing Burst song (marked with a “B”) were all Estrela 2S while those from males producing Pulse P5 songs (marked with a “P5”) were Estrela 1S. However, among the other sequences two interesting features are also observed in the network. Estrela 1S sequence Est1S13A clusters with Estrela 2S sequences while the other allele from the same fly (Est1S13B) clusters with other Estrela 1S sequences. The same happens to Estrela 1S sequences Est1S14A and Est1S14B; and Estrela 2S sequences Est2S24A and Est2S24B. This could indicate retention of ancestral polymorphisms, introgression or even possible hybrids between the two siblings. In addition, although the sequences Est2S30A and Est2S30B are the two alleles of one Estrela 2S fly both cluster with Estrela 1S sequences. This could indicate that the spot phenotype is not 100% reliable for identifying the two putative species in this locality.

**Figure 3:**
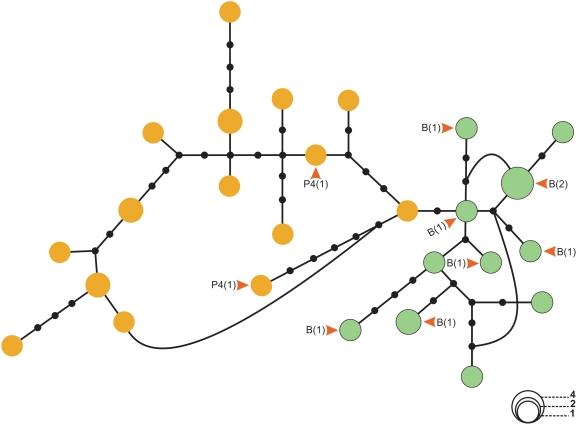
Haplotype network of the *L. longipalpis* sequences from Jaíba. Jaíba 1S (orange), Jaíba 2S (light green). Haplotypes from individuals with recorded copulation songs (B: Burst-type and P4: Pulse-type 4) and the number of sequences (in brackets) are shown. Curved lines represent alternative branching between haplotypes.

Our data show that Jaíba 1S and 2S morphotypes are also highly differentiated, as observed in Estrela de Alagoas and Sobral (Table 4, [26]). One fixed difference, 29 exclusive polymorphisms and only four shared polymorphic sites were observed between Jaíba 1S and 2S (Tables S2 and S3). The differentiation observed between the two Jaíba putative siblings is consistent with the song analysis (see above). Jaíba 2S shows very low and non-significant F_ST_ values when compared to Natal, Sobral 2S, Estrela 2S, Marajó and Pancas suggesting that all these populations represent essentially the same species (Table 4). Comparisons involving Jaíba 1S show a low and non-significant level of differentiation with Sobral 1S and Lapinha (Table 4). A haplotype network including all sequences from Jaíba (1S and 2S) is shown in Figure 3 (see also Figure S4 for a minimum evolution tree of the same sequences). The results are similar to Figure 2 and the network clearly separates Jaíba 1S and 2S sequences. Note also that the haplotypes from Jaíba 1S and 2S males that have had their songs analyzed (marked with “P4” and “B”, respectively) cluster consistently with the other sequences of their respective populations.

### Other samples and Global genealogy analysis

Table 4 shows that the lowest genetic differentiation involving Teresina was observed against Sobral 1S. On the other hand, the lowest and non-significant F_ST_ value observed for Pancas was against Sobral 2S. Pancas also showed low and non-significant values against Natal and Jaíba 2S, low but significant differentiation against Estrela 2S and moderate, but non-significant differentiation against Marajó. The latter is more closely related to other Burst song populations and showed higher levels of genetic differentiation when compared to Pulse song samples but all comparisons resulted in non-significant values, probably due to the small number of sequences available for this locality (Tables 3 and 4).

**Figure 4:**
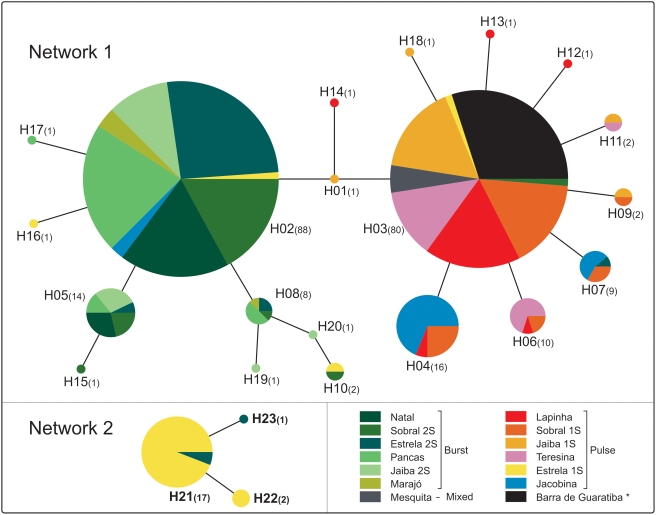
Haplotype networks of the *L. longipalpis* haplotypes from Brazil. Each *L. longipalpis* population is represented by one color. Each node (pie diagram) represents a unique haplotype (see Table S4). The number of sequences represented in each node is shown in brackets.

Figure 4 shows two haplotype networks based on a 58-bp non-recombinant segment within the 266-bp fragment. An attempt to construct a network based on the whole fragment resulted in too many ambiguities due to many recombination events (see also Bauzer *et al.* [25]) and therefore this smaller non-recombinant segment was used. Twenty-three haplotypes were identified with 18 segregating sites. Using a 95% connection limit, two networks were constructed. In network 1 (Figure 4 and Table S4) we found the two more frequent haplotypes (H2 and H3) connected to H1. The H2 is the predominant haplotype in Burst song populations of Natal, Sobral 2S, Estrela 2S, Pancas, Jaíba 2S and Marajó. On the other hand H3 is the predominant haplotype in the Pulse song populations of Lapinha, Sobral 1S, Jaíba 1S, and Teresina. Mesquita and Barra de Guaratiba show only the haplotype H3. The main haplotype found in Jacobina (H4) is connected to H3 by a single mutation. In network 2 we observed one major haplotype with 16 sequences of the Estrela 1S, and other two minor haplotypes with sequences of Estrela 1S and Estrela 2S (Figure 4 and Table S4). The two networks are connected if the significance level is decreased to 94%. In this case, the Estrela haplotype H21 is connected to H09 by two hypothetical haplotypes.

### *period* gene partition analysis according to copulation songs and pheromones

We examined the partitioning of *per* sequence variation within and between groups by performing an AMOVA. For this analysis, groups were defined based on copulation songs and pheromones (Table 5), the samples from Barra de Guaratiba and Mesquita were not included. Most of the total variation (over 50%) is distributed within populations in both analyses. For the pheromone groups based on the number of carbons (C16 and C20) or based on the different pheromone types (cembrene-1, cembrene-2, 9-methylgermacrene-B and himachalene), the variation was almost equally distributed between populations within the groups (18.73 and 22.90%, respectively) and among groups (23.25 and 18.37%, respectively). This result probably reflects the fact that Jaíba 1S and Jaíba 2S are both C20 but genetically quite different, and that Estrela 1S is cembrene-1 (and therefore C20) but genetically differentiated from the rest of these groups.

**Table 5:** AMOVA results for Brazilian populations of *L. longipalpis*.

Source of variation		
	Copulation songs	Pheromones
	Percentage of Variation	
Among groups	31.22 (40.20)	18.73 (22.90)
Among populations within groups	13.79 (1.68)	23.25 (18.37)
Within populations	54.99 (58.12)	58.02 (58.73)
	Fixation Indices	
*F_SC_* (haplotypes/populations within groups)	0.2005*** (0.0281***)	0.2861 (0.2383***)
*F_ST_* (haplotypes/populations/groups)	0.4501*** (0.4188***)	0.4198 (0.4127***)
*F_CT_* (populations/groups)	0.3122** (0.4019***)	0.1873 (0.2290**)

Copulation song Groups: Burst-type (Natal, Sobral 2S, Pancas, Estrela 2S, Jaíba 2S and Marajó), and Pulse-type (Jacobina - P1, Lapinha - P2, Teresina and Sobral 1S - P3, Jaíba 1S - P4 and Estrela 1S - P5). Results considering the different pulse-type songs separately are shown within brackets. Pheromone Groups: C20 (Estrela 2S and 1S, Jaíba 2S and 1S, Marajó, Pancas, Natal and Sobral 2S), and C16 (Teresina, Sobral 1S, Lapinha and Jacobina). Results considering pheromones Cembrene-1 (Estrela 2S and 1S, Jaíba 2S, Marajó, Pancas, Natal and Sobral 2S), Cembrene-2 (Jaíba 1S), Germacrene (Teresina, Sobral 1S and Lapinha) and Himachalene (Jacobina) separately are shown in brackets. Significance of fixation indices was evaluated by 10,000 random permutations (**: p<0.01; ***: p<0.001).

Considering the two main groups of copulation song patterns (Burst and Pulse), 13.79% of the total molecular variation is distributed among populations within groups. This variation is much smaller when we consider the different Pulse-type songs (P1, P2, P3, P4 and P5) as separate groups (1.68%) and reflects the high similarity among populations with Burst songs and between the two P3 populations (Sobral 1S and Teresina).

The low differentiation among Burst song populations contrasts to the differentiation among Pulse song populations (Table 4). Hence, the mean pairwise F_ST_ value among the Burst populations is only 0.058±0.017 while among samples of Pulse song populations it is 0.260±0.037. Finally, the differentiation among Pulse populations is nearly half of that observed for comparisons between the two song groups (mean pairwise F_ST_ = 0.449±0.011).

## Discussion

There are a number of difficulties associated with the study of recently diverged species and populations in an incipient speciation process [58]–[60] such as the members of the *L. longipalpis* complex within Brazil [10]. Although the role of sexual selection as a major cause of speciation still needs further support [61], it is likely that the rapid divergence of mating signals is particularly important in the evolution of reproductive isolation in cryptic species complexes [62]. Therefore, in order to enhance our knowledge on the taxonomic status and geographic distribution of the different *L. longipalpis* siblings in Brazil, we combined a comparison of *period* gene sequences with an analysis of male copulation songs and sex pheromones, traits that probably have an important role in the reproductive isolation among these closely related species.

Our results reveal a high level of complexity in the divergence and gene-flow among Brazilian populations of the *L. longipalpis* species complex. The available data suggest that the sibling species producing Burst-type copulation songs and cembrene-1 is distributed mainly throughout the coastal regions of North and Northeast Brazil reaching the Southeast in Pancas (Figure 1). In contrast, the populations producing different types of Pulse songs and pheromones are far more heterogeneous and probably represent five incipient species with different levels of divergence among the siblings.

The existence of pairs of sympatric species in three different localities (Sobral, Jaíba and Estrela de Alagoas) raises the question whether reinforcement of reproductive isolation [63] is occurring in this species complex as preliminary evidence suggests [42]. In each case a Burst song, cembrene-1 population is sympatric to a different Pulse song sibling. In Sobral, the two siblings also differ in their pheromone types, and their genetic differentiation and reproductive isolation have been confirmed by microsatellites [28], the *cacophony* gene [29] and crossing experiments [14],[15],[42]. In Jaíba, the two siblings also differ in the type of diterpene isomers they carry [53]. Finally, Estrela 1S and 2S males differ only in their copulation songs, but they share the same type of pheromone [38]. Interestingly though, Estrela 1S is the most genetically divergent among the Pulse-type populations. Acoustic signals and pheromones are both likely to have a role in the reproductive isolation of the *L. longipalpis* siblings as observed in other insects [64]. The pheromones are probably involved in pre-mating isolation [42] while copulation songs might be the main signal involved in the insemination failure observed in copulations between the siblings [14],[15],[42]. The fact that in Estrela, males of the two siblings have the same pheromone suggests that in this locality copulation songs might have a more important role as isolation mechanism or, that other signals, such as, visual or putative cuticular pheromones are also involved [65]. Our results with Estrela also show how important is to carry out an integrative approach using molecular and behavioral data as the analysis of pheromone alone in this locality would suggest a single species. It is possible that future work will reveal siblings that differ in pheromones but not in their copulation songs.

Besides the Burst and Pulse songs, analysis of the acoustic signals produced by Mesquita males revealed a new pattern that we called Mix because it presents characteristics that resemble superficially the two other types. The trains of the Mix song begin like a polycyclic Pulse song and end with a more Burst-like pattern. Interestingly, the switch from one pattern to the other in the Mesquita song is also associated with characteristic changes in male behavior. Males that produce Burst song not only vibrate their wings but also swing their bodies continuously about 30° degrees to each side during copulation. Males producing Pulse songs do not do that. Mesquita males produce songs in both ways. They start the copulation like Pulse song males and then begin swinging their bodies the same way Burst song males do (Vigoder and Peixoto, unpublished observations). Unfortunately the Mesquita sample we analyzed was very small and new samples from the same collection site are impossible to obtain at the moment. We are currently attempting to obtain further samples from other localities in Rio de Janeiro State, a region where *L. longipalpis* is usually quite rare. Analysis of other populations producing this new song pattern might offer interesting clues about the evolution of the copulation songs and the speciation in the *L. longipalpis* complex.

Barra de Guaratiba is also an interesting population because although males from this locality produce mainly the 9-methylgermacrene pheromone, large amounts of cembrenes are also found in their extracts. We observed that Barra de Guaratiba is a monomorphic population for the 266 bp fragment of the *per* gene analyzed. This lack of molecular variation is consistent with a very small population size. However, a recent bottleneck or a selective sweep event in or near the *per* locus with fixation of a single haplotype are also possible alternative explanations. Analysis of a different gene is needed to settle the issue.

Our data show evidence for the persistence of ancestral polymorphisms and/or introgression, suggesting that the separation between the Brazilian siblings is probably recent and perhaps incomplete as suggested by crossing experiments [14],[15]. Differential introgression across the genome might be occurring in *L. longipalpis* causing a mosaic of genetic divergence as observed in other sand flies [66] and in the *Anopheles gambiae* complex and *An. gambiae s.s.* incipient species [67]–[71]. That is consistent with the fact that different conclusions concerning the taxonomic status of *L. longipalpis* in Brazil were reached by studies using different genetic markers (reviewed in [10]).

Introgression between closely related or incipient vector species can have very important epidemiological consequences allowing the spread of insecticide resistance genes and adaptive traits as well as facilitating the bridge between sylvatic and peri-urban cycles [66]–[68],[72],[73]. In addition, man made environmental changes might promote contact between different incipient species with incomplete reproductive isolation that will in turn exchange reservoirs of genetic variability that might promote adaptation to modified habitats. Whether this could be one of the explanations for the spread of visceral leishmaniasis observed in the last decades in Brazil [5], is still an open question. To address the issue of introgression between the *L. longipalpis* siblings in Brazil in more detail, we are currently carrying out a multilocus analysis of the sympatric siblings from Sobral that we hope will help understanding the mechanisms shaping the differentiation in this complex. Extending our studies to other populations will also give us a better view of the geographical distribution of the *L. longipalpis* sibling species in Brazil and their potential implication to Leishmania transmission.

## Supporting Information

Figure S1 Male copulation song traces from the populations analyzed in this study. One second samples are shown in all cases except for the bottom right trace that shows a complete train of a Mesquita male song.
(2.30 MB TIF)

Figure S2 Variable sites. In gray the variable sites in the intron region. (*) sequences with non-silent substitutions (in italic).
(0.43 MB TIF)

Figure S3 Minimum Evolution tree of the *L. longipalpis* sequences from Estrela de Alagoas. Only bootstrap values above 50% are shown and they are based on 2,000 replications. Estrela 1S (yellow squares), Estrela 2S (green squares). As observed for the haplotype network, the sequences are separated in two major groups. The first one consists mainly of Estrela 1S sequences, plus three haplotypes of Estrela 2S. The second group consists of Estrela 2S sequences, plus three haplotypes of Estrela 1S. The sequences from males producing Burst songs are marked with a “*B”, whereas those from males producing Pulse P5 songs are marked with a “*P5”.
(1.27 MB TIF)

Figure S4 Minimum Evolution tree of the *L. longipalpis* sequences from Jaíba. Only bootstrap values above 50% are shown and they are based on 2,000 replications. Jaíba 1S (orange circles), Jaíba 2S (light green circles). As in the haplotype network, this tree clearly separates Jaíba 1S and 2S sequences. The sequences from males that have had their songs analyzed (marked with “*P4” and “*B”, respectively) cluster consistently with the other sequences of their respective populations.
(0.91 MB TIF)

Table S1 Analysis of variance comparing the two parts of the Mix type song and each segment with the populations with similar song type.
(0.03 MB DOC)

Table S2 Shared (upper right matrix) and fixed (lower left matrix) polymorphic sites.
(0.05 MB DOC)

Table S3 Number of exclusive sites in population X (upper right matrix) and Y (lower left matrix).
(0.05 MB DOC)

Table S4 Distribution of 23 haplotypes among *L. longipalpis* samples, segregating sites within a 58-bp non-recombinant fragment and number of sequences represented in each sample.
(0.11 MB DOC)

Alternative Language Abstract S1 Translation of the abstract into Portuguese by Luiz G. S. R. Bauzer
(0.01 MB PDF)

Alternative Language Abstract S2 Translation of the abstract into Spanish by Alejandra Saori Araki
(0.01 MB PDF)

## References

[1] Bickford D, Lohman DJ, Sodhi NS, Ng PK, Meier R (2007). Cryptic species as a window on diversity and conservation.. Trends Ecol Evol.

[2] Ayala FJ, Coluzzi M (2005). Chromosome speciation: humans, Drosophila, and mosquitoes.. Proc Natl Acad Sci U S A.

[3] Young DG, Duncan MA (1994). Guide to the identification and geographic distribution of *Lutzomyia* sand flies in Mexico, the West Indies, Central and South America (Diptera: Psychodidae).. Mem Amer Ent Inst.

[4] Soares RP, Turco SJ (2003). *Lutzomyia longipalpis* (Diptera: Psychodidae: Phlebotominae): a review.. An Acad Bras Cienc.

[5] Lainson R, Rangel EF (2005). *Lutzomyia longipalpis* and the eco-epidemiology of American visceral leishmaniasis, with particular reference to Brazil: a review.. Mem Inst Oswaldo Cruz.

[6] Lanzaro GC, Ostrovska K, Herrero MV, Lawyer PG, Warburg A (1993). *Lutzomyia longipalpis* is a species complex: genetic divergence and interspecific hybrid sterility among three populations.. Am J Trop Med Hyg.

[7] Lanzaro GC, Warburg A (1995). Genetic variability in phlebotomine sandflies: possible implications for leishmaniasis epidemiology.. Parasitol Today.

[8] Munstermann LE, Morrison AC, Ferro C, Pardo R, Torres M (1998). Genetic structure of local populations of *Lutzomyia longipalpis* (Diptera: Psychodidae) in Central Colombia.. J Med Entomol.

[9] Arrivillaga J, Mutebi JP, Pinango H, Norris D, Alexandre B (2003). The taxonomic status of genetically divergent populations of *Lutzomyia longipalpis* (Diptera: Psychodidae) based on the distribution of mitochondrial and isozyme variation.. J Med Entomol.

[10] Bauzer LG, Souza NA, Maingon RDC, Peixoto AA (2007). *Lutzomyia longipalpis* in Brazil: a complex or a single species? A mini-review.. Mem Inst Oswaldo Cruz.

[11] Conn JE, Mirabello L (2007). The biogeography and population genetics of neotropical vector species.. Heredity.

[12] Coutinho-Abreu IV, Sonoda IV, Fonseca JA, Melo MA, Balbino VQ (2008). *Lutzomyia longipalpis* s.l. in Brazil and the impact of the Sao Francisco River in the speciation of this sand fly vector.. Parasit Vectors.

[13] Mangabeira O (1969). Sobre a sistemática e biologia dos flebótomos do Ceará.. Rev Bras Malarial D Trop.

[14] Ward RD, Ribeiro AL, Ready PD, Murtagh A (1983). Reproductive isolation between different forms of *Lutzomyia longipalpis* (Lutz & Neiva, 1912) (Diptera: Psychodidae) the vector of *Leishmania donovani chagasi* Cunha & Chagas and its significance to Kala-azar distribution in South America.. Mem Inst Oswaldo Cruz.

[15] Ward RD, Phillips A, Burnet B, Marcondes CB, Service MW (1988). The *Lutzomyia longipalpis* complex: reproduction and distribution.. Biosystematics of Haematophagous Insects.

[16] Maingon RD, Ward RD, Hamilton JG, Bauzer LG, Peixoto AA (2008). The *Lutzomyia longipalpis* species complex: does population sub-structure matter to *Leishmania* transmission?. Trends Parasitol.

[17] Lambrechts L, Halbert J, Durand P, Gouagna LC, Koella JC (2005). Host genotype by parasite genotype interactions underlying the resistance of anopheline mosquitoes to *Plasmodium falciparum*.. Malar J.

[18] Kyriacou CP, Hall JC (1986). Interspecific genetic control of courtship song production and reception in *Drosophila*.. Science.

[19] Konopka RJ, Benzer S (1971). Clock mutants of *Drosophila melanogaster*.. Proc Natl Acad Sci U S A.

[20] Kyriacou CP, Hall JC (1982). The function of courtship song rhythms in *Drosophila*.. Anim Behav.

[21] Wheeler DA, Kyriacou CP, Greenacre ML, Yu Q, Rutila JE (1991). Molecular transfer of a species-specific behavior from *Drosophila simulans* to *Drosophila melanogaster*.. Science.

[22] Ritchie MG, Halsey EJ, Gleason JM (1999). *Drosophila* song as a species-specific mating signal and the behavioural importance of Kyriacou & Hall cycles in *D. melanogaster* song.. Anim Behav.

[23] Coyne JA (1992). Genetics and speciation.. Nature.

[24] Peixoto AA (2002). Evolutionary behavioral genetics in *Drosophila*.. Adv Genet.

[25] Bauzer LG, Souza NA, Ward RD, Kyriacou CP, Peixoto AA (2002a). The *period* gene and genetic differentiation between three Brazilian populations of *Lutzomyia longipalpis*.. Insect Mol Biol.

[26] Bauzer LG, Gesto JS, Souza NA, Ward RD, Hamilton JG (2002b). Molecular divergence in the *period* gene between two putative sympatric species of the *Lutzomyia longipalpis* complex.. Mol Biol Evol.

[27] Watts PC, Hamilton JG, Ward RD, Noyes HA, Souza NA (2005). Male sex pheromones and the phylogeographic structure of the *Lutzomyia longipalpis* species complex (Diptera: Psychodidae) from Brazil and Venezuela.. Am J Trop Med Hyg.

[28] Maingon RD, Ward RD, Hamilton JG, Noyes HA, Souza N (2003). Genetic identification of two sibling species of *Lutzomyia longipalpis* (Diptera: Psychodidae) that produce distinct male sex pheromones in Sobral, Ceará State, Brazil.. Mol Ecol.

[29] Bottecchia M, Oliveira SG, Bauzer LG, Souza NA, Ward RD (2004). Genetic divergence in the *cacophony* IVS6 intron among five Brazilian populations of *Lutzomyia longipalpis*.. J Mol Evol.

[30] Ewing AW (1989). Arthropod Bioacoustics: Neurobiology and Behaviour.

[31] Ritchie MG, Gleason JM (1995). Rapid evolution of courtship song pattern in *Drosophila willistoni* sibling species.. J Evol Biol.

[32] Souza NA, Vigoder FM, Araki AS, Ward RD, Kyriacou CP (2004). Analysis of the copulatory courtship songs of *Lutzomyia longipalpis* in six populations from Brazil.. J Med Entomol.

[33] de Souza NA, Ward RD, Hamilton JG, Kyriacou CP, Peixoto AA (2002). Copulation songs in three siblings of *Lutzomyia longipalpis* (Diptera: Psychodidae).. Trans R Soc Trop Med Hyg.

[34] Hall JC (1994). The mating of a fly.. Science.

[35] Hoikkala A, Crossley S (2000). Copulatory courtship in Drosophila: behavior and songs of *D. birchii* and *D. serrata*.. J Insect Behav.

[36] Hamilton JG, Hooper AM, Mori K, Pickett JA, Sano A (1999a). 3-Methyl-α-himachalene confirmed, and the relative stereochemistry defined, by synthesis as the sex pheromone of the sandfly *Lutzomyia longipalpis* from Jacobina, Brazil.. Chem Commun.

[37] Hamilton JG, Ibbotson HC, Hooper AM, Mori K, Picket JA (1999b). 9-Methylgermacrene-B confirmed by synthesis as the sex pheromone of the sandfly *Lutzomyia longipalpis* from Lapinha, Brazil, and the absolute stereochemistry defined as 9S.. Chem Commun.

[38] Hamilton JG, Maingon RD, Alexander B, Ward RD, Brazil RP (2005). Analysis of the sex pheromone extract of individual male *Lutzomyia longipalpis* sandflies from six regions in Brazil.. Med Vet Entomol.

[39] Spiegel CN, Jeanbourquin P, Guerin PM, Hooper AM, Claude S (2005). (1S,3S,7R)-3-methyl-alpha-himachalene from the male sandfly *Lutzomyia longipalpis* (Diptera: Psychodidae) induces neurophysiological responses and attracts both males and females.. J Insect Physiol.

[40] Ward RD, Morton IE (1991). Pheromones in mate choice and sexual isolation between siblings of *Lutzomyia longipalpis* (Diptera:Psychodidae).. Parassitologia.

[41] Morton IE, Ward RD (1989). Laboratory response of female *Lutzomyia longipalpis* sandflies to a host and male pheromone source over distance.. Med Vet Entomol.

[42] Souza NA, Andrade-Coelho CA, Vigoder FM, Ward RD, Peixoto AA (2008). Reproductive isolation between sympatric and allopatric Brazilian populations of *Lutzomyia longipalpis s.l.* (Diptera: Psychodidae).. Mem Inst Oswaldo Cruz.

[43] Jowett T, Roberts DB (1998). Preparation of nucleic acids.. *Drosophila*: A practical approach.

[44] Sambrook D, Russell J (2001). Molecular cloning: a laboratory manual.

[45] Rozas J, Rozas R (1999). DnaSP version 3: An integrated program for molecular population genetics and molecular evolution analysis.. Bioinformatics.

[46] Filatov DA, Charlesworth D (1999). DNA polymorphism, haplotype structure and balancing selection in the Leavenworthia PgiC locus.. Genetics.

[47] Kumar S, Tamura K, Nei M (2004). MEGA3: Integrated Software for Molecular Evolutionary Genetics Analysis and Sequence Alignment.. Brief Bioinform.

[48] Excoffier L, Laval G, Schneider S (2005). Arlequin ver 3.0: An integrated software package for population genetics data analysis.. Evol Bioinformatics Online.

[49] Hudson RR, Kaplan NL (1985). Statistical Properties of the Number of Recombination Events in the History of a Sample of DNA Sequences.. Genetics.

[50] Templeton AR, Crandall KA, Sing CF (1992). A cladistic analysis of phenotypic associations with haplotypes inferred from restriction endonuclease mapping and DNA sequence data. III. Cladogram estimation.. Genetics.

[51] Clement M, Posada D, Crandall KA (2000). TCS: a computer program to estimate gene genealogies.. Mol Ecology.

[52] Gorczyca MG, Hall JC (1987). The INSECTAVOX, an integrated device for recording and amplifying courtship songs of *Drosophila*.. Dros Inf Serv.

[53] Hamilton JG, Brazil RP, Maingon R (2004). A fourth chemotype of *Lutzomyia longipalpis* (Diptera: Psychodidae) from Jaibas, Minas Gerais State, Brazil.. J Med Entomol.

[54] Tajima F (1989). Statistical method for testing the neutral mutation hypothesis by DNA polymorphism.. Genetics.

[55] Fu YX (1997). Statistical test of neutrality of mutations against population growth, hitchhiking and background selection.. Genetics.

[56] Ramos-Onsins SE, Rozas J (2002). Statistical properties of new neutrality tests against population growth.. Mol Biol Evol.

[57] Hudson RR, Slatkin M, Maddison WP (1992). Estimation of levels of gene flow from DNA sequence data.. Genetics.

[58] Hey J (2001). Genes, Categories, and Species.

[59] Hey J, Machado CA (2003). The study of structured populations – new hope for a difficult and divided science.. Nat Rev Genet.

[60] Coyne JA, Orr HA (2004). Speciation.

[61] Ritchie MG (2007). Sexual Selection and Speciation.. Ann Rev Ecol Evol Syst.

[62] Mendelson TC, Shaw KL (2005). Sexual behaviour: rapid speciation in an arthropod.. Nature.

[63] Servedio MR (2004). The what and why of research on reinforcement.. PLoS Biol.

[64] Mullen SP, Mendelson TC, Schal C, Shaw KL (2007). Rapid evolution of cuticular hydrocarbons in a species radiation of acoustically diverse Hawaiian crickets (Gryllidae: Trigonidiinae: Laupala).. Evolution.

[65] Bray DP, Hamilton JG (2007). Courtship behaviour in the sandfly *Lutzomyia longipalpis*, the New World vector of visceral leishmaniasis.. Med Vet Entomol.

[66] Mazzoni CJ, Araki AS, Ferreira GE, Azevedo RV, Barbujani G (2008). Multilocus analysis of introgression between two sand fly vectors of leishmaniasis.. BMC Evol Biol.

[67] Besansky NJ, Krzywinski J, Lehmann T, Simard F, Kern M (2003). Semipermeable species boundaries between *Anopheles gambiae* and *Anopheles arabiensis*: Evidence from multilocus DNA sequence variation.. Proc Natl Acad Sci U S A.

[68] Slotman MA, Della Torre A, Calzetta M, Powell JR (2005). Differential introgression of chromosomal regions between *Anopheles gambiae* and *An. arabiensis*.. Am J Trop Med Hyg.

[69] Stump AD, Fitzpatrick MC, Lobo NF, Traoré S, Sagnon N (2005). Centromere-proximal differentiation and speciation in *Anopheles gambiae*.. Proc Natl Acad Sci U S A.

[70] Tripet F, Dolo G, Lanzaro GC (2005). Multilevel analyses of genetic differentiation in *Anopheles gambiae s.s.* reveal patterns of gene flow important for malaria-fighting mosquito projects.. Genetics.

[71] Wang-Sattler R, Blandin S, Ning Y, Blass C, Dolo G (2007). Mosaic Genome Architecture of the *Anopheles gambiae* Species Complex.. PLoS ONE.

[72] Fonseca DM, Keyghobadi N, Malcolm CA, Mehmet C, Schaffner F (2004). Emerging vectors in the *Culex pipiens* complex.. Science.

[73] Djogbénou L, Chandre F, Berthomieu A, Dabiré R, Koffi A (2008). Evidence of introgression of the *ace-1^R^* mutation and of the *ace-1* duplication in West African *Anopheles gambiae s. s.*. PLoS ONE.

