# Supplementary figures and images for: Molecular and Behavioral Differentiation among Brazilian Populations of Lutzomyia longipalpis (Diptera: Psychodidae: Phlebotominae)
Source: PLoS Negl Trop Dis. 2009 Jan 27;3(1):e365. doi: 10.1371/journal.pntd.0000365 (PMC2628317; doi:10.1371/journal.pntd.0000365)

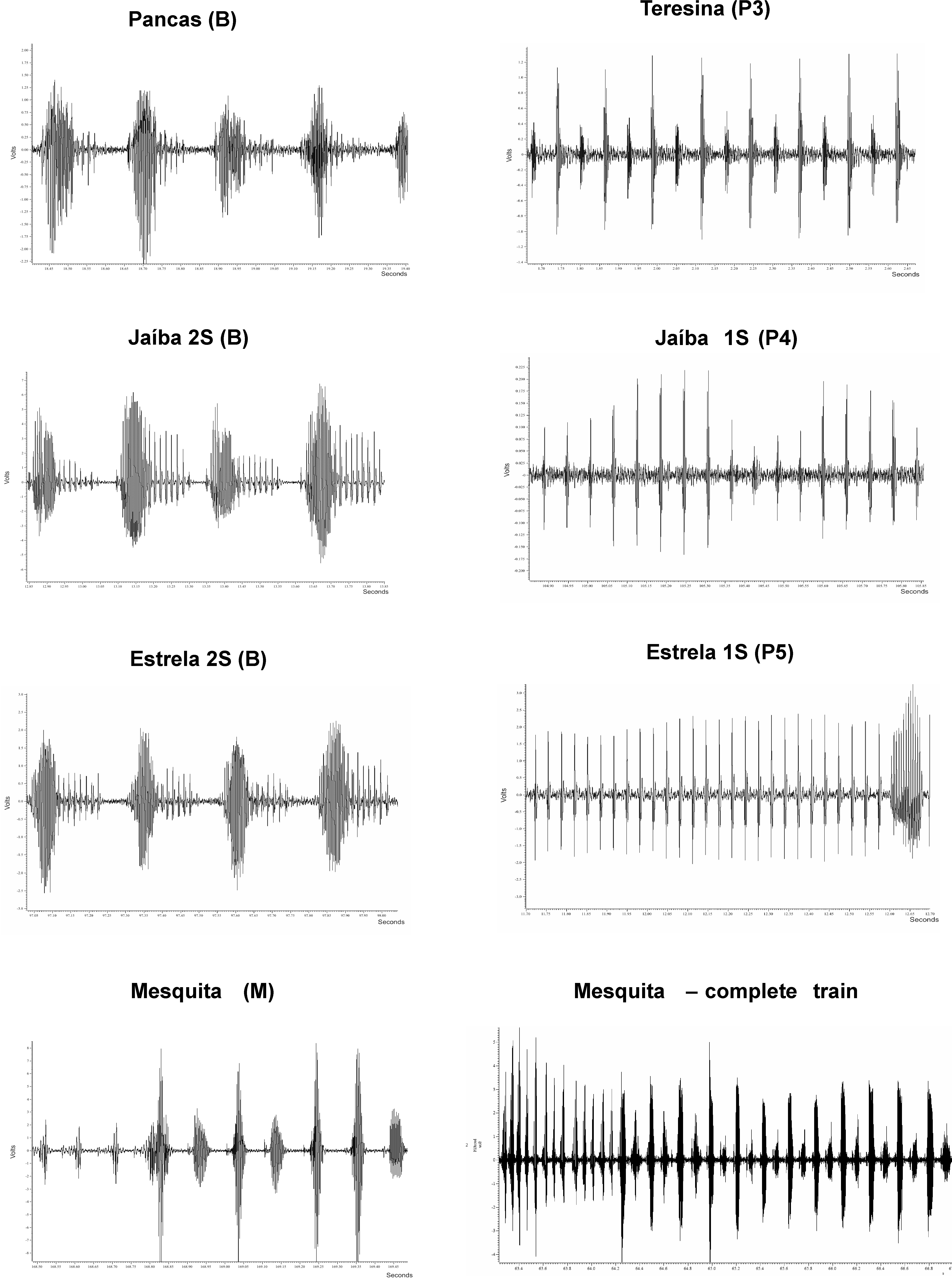

Supplement: Figure S1 — Male copulation song traces from the populations analyzed in this study. One second samples are shown in all cases except for the bottom right trace that shows a complete train of a Mesquita male song. (2.30 MB TIF) [file pntd.0000365.s001.tif]

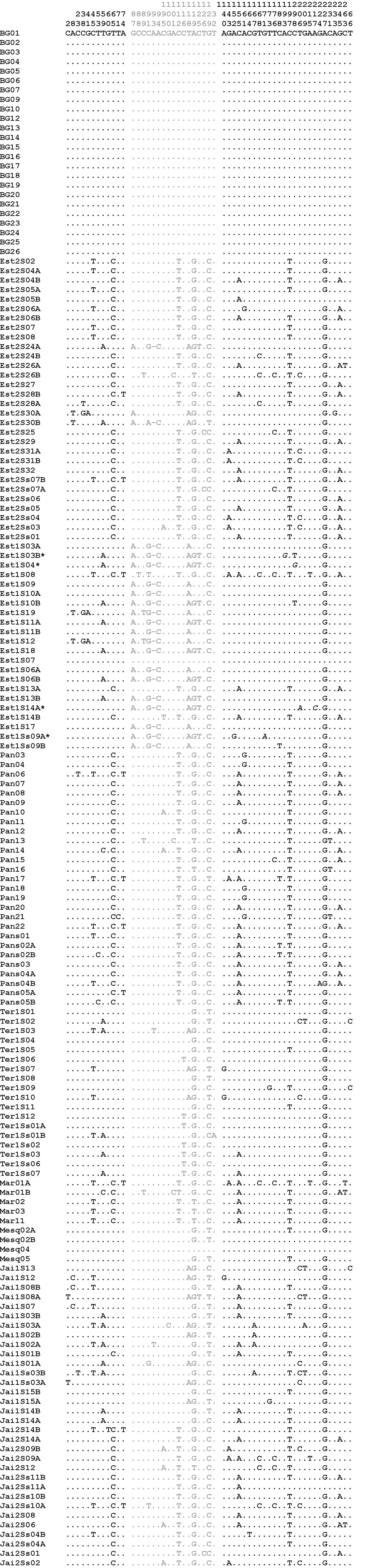

Supplement: Figure S2 — Variable sites. In gray the variable sites in the intron region. (*) sequences with non-silent substitutions (in italic). (0.43 MB TIF) [file pntd.0000365.s002.tif]

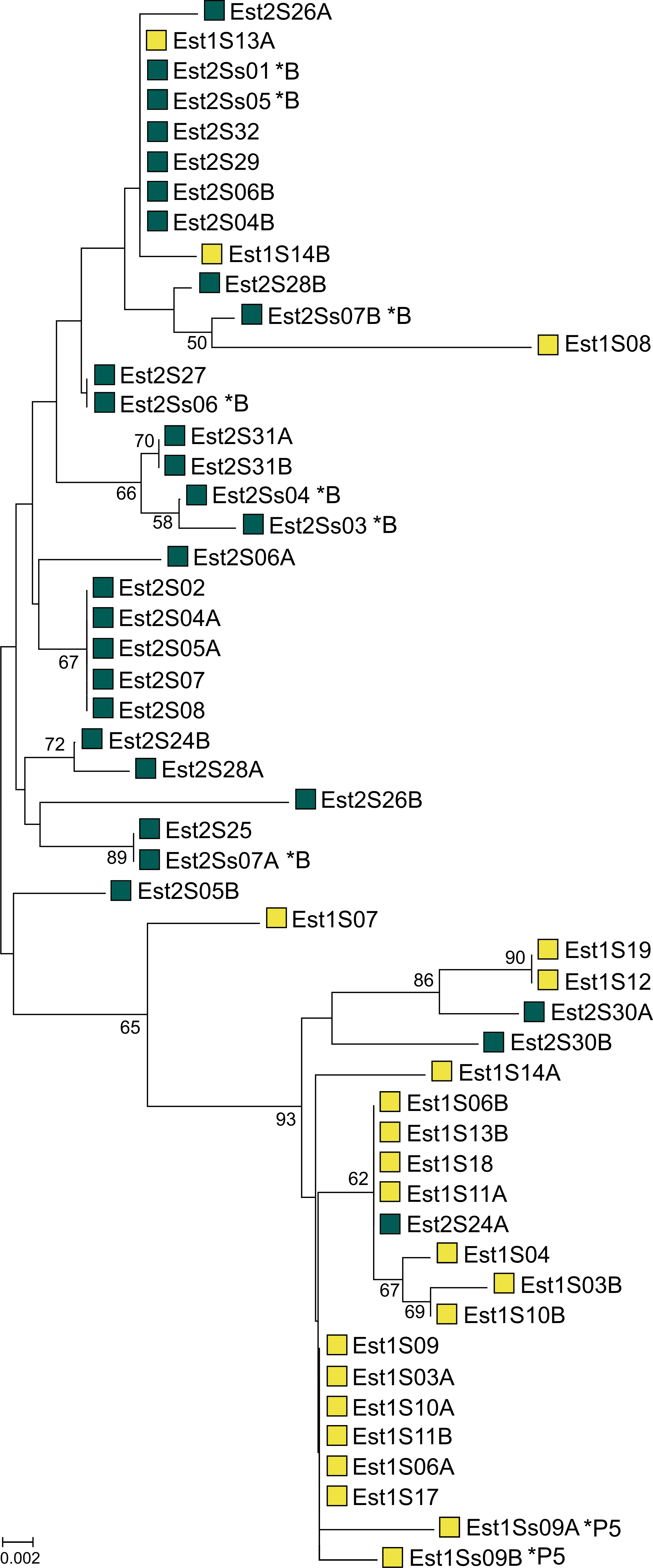

Supplement: Figure S3 — Minimum Evolution tree of the L. longipalpis sequences from Estrela de Alagoas. Only bootstrap values above 50% are shown and they are based on 2,000 replications. Estrela 1S (yellow squares), Estrela 2S (green squares). As observed for the haplotype network, the sequences are separated in two major groups. The first one consists mainly of Estrela 1S sequences, plus three haplotypes of Estrela 2S. The second group consists of Estrela 2S sequences, plus three haplotypes of Estrela 1S. The sequences from males producing Burst songs are marked with a “*B”, whereas those from males producing Pulse P5 songs are marked with a “*P5”. (1.27 MB TIF) [file pntd.0000365.s003.tif]

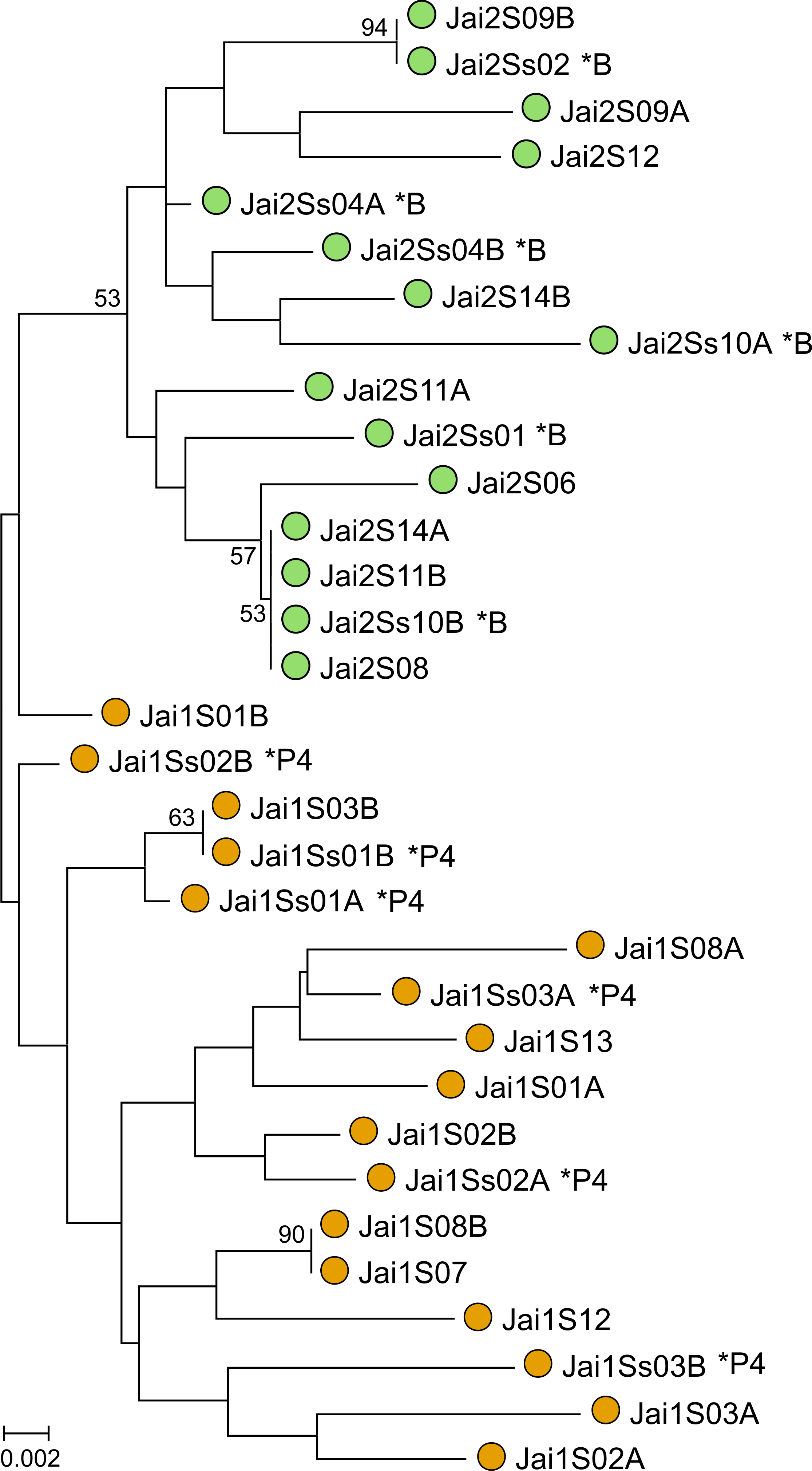

Supplement: Figure S4 — Minimum Evolution tree of the L. longipalpis sequences from Jaíba. Only bootstrap values above 50% are shown and they are based on 2,000 replications. Jaíba 1S (orange circles), Jaíba 2S (light green circles). As in the haplotype network, this tree clearly separates Jaíba 1S and 2S sequences. The sequences from males that have had their songs analyzed (marked with “*P4” and “*B”, respectively) cluster consistently with the other sequences of their respective populations. (0.91 MB TIF) [file pntd.0000365.s004.tif]
