# Supplementary material for: Molecular and Behavioral Differentiation among Brazilian Populations of Lutzomyia longipalpis (Diptera: Psychodidae: Phlebotominae)
Source: PLoS Negl Trop Dis. 2009 Jan 27;3(1):e365. doi: 10.1371/journal.pntd.0000365 (PMC2628317; doi:10.1371/journal.pntd.0000365)
Supplement: Table S1 — Analysis of variance comparing the two parts of the Mix type song and each segment with the populations with similar song type. (0.03 MB DOC) [file pntd.0000365.s005.doc]

**Supplemental TABLE S1**

**Analysis of variance comparing the two parts of the Mix type song and each segment with**

**the populations with similar song type.**

| **Comparison** | **IPI/IBI** | **NP** | **TL** | **Freq** | **CPP** |
| --- | --- | --- | --- | --- | --- |
| **Between the two segments** | F_(1,6)_ = 14.25* | F_(1,6)_ = 114.11** | F_(1,6)_ = 69.28** | F_(1,6)_ = 256.25** | F_(1,6)_ = 204.94** |
| **First segment with the Pulse-type populations** | F_(6,50)_ = 46.60** | F_(6,50)_ = 58.65** | F_(6,50)_ = 19.76** | F_(6,50)_ = 35.51** | F_(6,50)_ = 100.65** |
| **Second segment with the Burst-type populations** | F_(8,52)_ = 12.70** | F_(8,52)_ = 22.33** | F_(8,52)_ = 1.23^ns^ | F_(8,52)_ = 2.82* |  |

*P<0.01; ** P<0.001; ^ns^ non significant
