# Supplementary material for: Molecular and Behavioral Differentiation among Brazilian Populations of Lutzomyia longipalpis (Diptera: Psychodidae: Phlebotominae)
Source: PLoS Negl Trop Dis. 2009 Jan 27;3(1):e365. doi: 10.1371/journal.pntd.0000365 (PMC2628317; doi:10.1371/journal.pntd.0000365)
Supplement: Table S2 — Shared (upper right matrix) and fixed (lower left matrix) polymorphic sites. (0.05 MB DOC) [file pntd.0000365.s006.doc]

**Supplemental TABLE S2.**

**Shared (upper right matrix) and fixed (lower left matrix) polymorphic sites.**

| **Ss**  **Sf** | **E1S** | **J1S** | **Ter** | **Jac** | **Lap** | **S1S** | **E2S** | **J2S** | **Mar** | **Pan** | **Nat** | **S2S** |
| --- | --- | --- | --- | --- | --- | --- | --- | --- | --- | --- | --- | --- |
| **E1S** |  | 7 | 5 | 13 | 6 | 12 | 19 | 11 | 8 | 9 | 10 | 15 |
| **J1S** | 0 |  | 11 | 6 | 12 | 15 | 0 | 4 | 1 | 4 | 4 | 7 |
| **Ter** | 0 | 0 |  | 4 | 11 | 11 | 0 | 2 | 2 | 1 | 4 | 5 |
| **Jac** | 0 | 0 | 0 |  | 6 | 11 | 0 | 0 | 0 | 0 | 9 | 10 |
| **Lap** | 0 | 0 | 0 | 0 |  | 14 | 0 | 1 | 1 | 0 | 4 | 6 |
| **S1S** | 0 | 0 | 0 | 0 | 0 |  | 0 | 1 | 1 | 0 | 1 | 6 |
| **E2S** | 0 | 9 | 7 | 16 | 8 | 14 |  | 13 | 9 | 12 | 13 | 15 |
| **J2S** | 0 | 1 | 4 | 6 | 4 | 4 | 0 |  | 9 | 8 | 12 | 13 |
| **Mar** | 0 | 1 | 1 | 6 | 1 | 1 | 0 | 0 |  | 9 | 9 | 9 |
| **Pan** | 0 | 0 | 3 | 10 | 3 | 3 | 0 | 0 | 0 |  | 10 | 12 |
| **Nat** | 0 | 1 | 2 | 0 | 1 | 5 | 0 | 0 | 0 | 0 |  | 14 |
| **S2S** | 0 | 0 | 0 | 0 | 0 | 0 | 0 | 0 | 0 | 0 | 0 |  |

E1S: Estrela 1S, J1S: Jaíba 1S, Ter: Teresina, Jac: Jacobina, Lap: Lapinha, S1S: Sobral 1S, E2S: Estrela 2S, J2S: Jaíba 2S, Mar: Marajó, Pan: Pancas, Nat: Natal, S2S: Sobral 2S.
