# Supplementary material for: Molecular and Behavioral Differentiation among Brazilian Populations of Lutzomyia longipalpis (Diptera: Psychodidae: Phlebotominae)
Source: PLoS Negl Trop Dis. 2009 Jan 27;3(1):e365. doi: 10.1371/journal.pntd.0000365 (PMC2628317; doi:10.1371/journal.pntd.0000365)
Supplement: Table S3 — Number of exclusive sites in population X (upper right matrix) and Y (lower left matrix). (0.05 MB DOC) [file pntd.0000365.s007.doc]

**Supplemental TABLE S3.**

**Number of exclusive sites in population X (upper right matrix) and Y (lower left matrix).**

| **Sy**  **Sx** | **E1S** | **J1S** | **Ter** | **Jac** | **Lap** | **S1S** | **E2S** | **J2S** | **Mar** | **Pan** | **Nat** | **S2S** |
| --- | --- | --- | --- | --- | --- | --- | --- | --- | --- | --- | --- | --- |
| **E1S** |  | 28 | 24 | 22 | 29 | 23 | 10 | 24 | 27 | 26 | 25 | 20 |
| **J1S** | 12 |  | 2 | 14 | 7 | 4 | 10 | 14 | 18 | 15 | 15 | 12 |
| **Ter** | 8 | 8 |  | 15 | 2 | 2 | 6 | 9 | 10 | 10 | 13 | 8 |
| **Jac** | 7 | 13 | 9 |  | 14 | 9 | 3 | 14 | 14 | 10 | 11 | 10 |
| **Lap** | 13 | 7 | 8 | 13 |  | 5 | 11 | 15 | 18 | 16 | 15 | 13 |
| **S1S** | 16 | 13 | 16 | 17 | 14 |  | 13 | 24 | 27 | 25 | 23 | 22 |
| **E2S** | 10 | 20 | 22 | 13 | 21 | 15 |  | 16 | 20 | 17 | 16 | 14 |
| **J2S** | 7 | 15 | 13 | 12 | 14 | 14 | 4 |  | 9 | 10 | 6 | 5 |
| **Mar** | 4 | 11 | 12 | 6 | 11 | 11 | 2 | 3 |  | 11 | 3 | 3 |
| **Pan** | 11 | 16 | 16 | 10 | 17 | 17 | 7 | 12 | 3 |  | 10 | 8 |
| **Nat** | 8 | 16 | 9 | 9 | 14 | 13 | 4 | 6 | 9 | 8 |  | 4 |
| **S2S** | 7 | 15 | 17 | 12 | 16 | 16 | 7 | 9 | 13 | 10 | 8 |  |

E1S: Estrela 1S, J1S: Jaíba 1S, Ter: Teresina, Jac: Jacobina, Lap: Lapinha, S1S: Sobral 1S, E2S: Estrela 2S, J2S: Jaíba 2S, Mar: Marajó, Pan: Pancas, Nat: Natal, S2S: Sobral 2S.
