# Supplementary material for: Molecular and Behavioral Differentiation among Brazilian Populations of Lutzomyia longipalpis (Diptera: Psychodidae: Phlebotominae)
Source: PLoS Negl Trop Dis. 2009 Jan 27;3(1):e365. doi: 10.1371/journal.pntd.0000365 (PMC2628317; doi:10.1371/journal.pntd.0000365)
Supplement: Table S4 — Distribution of 23 haplotypes among L. longipalpis samples, segregating sites within a 58-bp non-recombinant fragment and number of sequences represented in each sample. (0.11 MB DOC) [file pntd.0000365.s008.doc]

**Supplemental TABLE S4**

**Distribution of 23 haplotypes among *L. longipalpis* samples, segregating sites within a 58-bp non-recombinant fragment and number of sequences represented in each sample.**

|  |  | Segregating sites | Number of sequences represented in each sample | | | | | | | | | | | | | | |
| --- | --- | --- | --- | --- | --- | --- | --- | --- | --- | --- | --- | --- | --- | --- | --- | --- | --- |
|  | Haplotype | 111223333344555 | BG | E1S | E2S | J1S | J2S | Mar | Mes | Pan | Ter | Jac | Lap | Nat | S1S | S2S | Total |
|  |  | 169258890245617137 |  |  |  |  |  |  |  |  |  |  |  |  |  |  |  |
| Network  1 | H01 | GCTTAAGCCCAACGCACC |  |  |  | 1 |  |  |  |  |  |  |  |  |  |  | 1 |
|  | H02 | ................T. |  | 1 | 23 |  | 9 | 3 |  | 19 |  | 2 |  | 16 |  | 15 | 88 |
|  | H03 | .T................ | 24 | 1 |  | 13 |  |  | 4 |  | 10 |  | 14 |  | 13 | 1 | 80 |
|  | H04 | .T...T............ |  |  |  |  |  |  |  |  |  | 11 | 1 |  | 4 |  | 16 |
|  | H05 | ............A...T. |  |  | 1 |  | 4 |  |  | 2 |  |  |  | 4 |  | 3 | 14 |
|  | H06 | .T...............T |  |  |  |  |  |  |  |  | 7 |  | 1 |  | 2 |  | 10 |
|  | H07 | .T....A........... |  |  | 1 |  |  |  |  |  |  | 5 |  |  | 3 |  | 9 |
|  | H08 | ....T...........T. |  |  | 2 |  |  | 1 |  | 4 |  |  |  |  |  | 1 | 8 |
|  | H09 | .T.......G........ |  |  |  | 1 |  |  |  |  |  |  |  |  | 1 |  | 2 |
|  | H10 | ....T..T.T......T. |  | 1 |  |  |  |  |  |  |  |  |  |  |  | 1 | 2 |
|  | H11 | .T........T....... |  |  |  | 1 |  |  |  |  | 1 |  |  |  |  |  | 2 |
|  | H12 | .T............T... |  |  |  |  |  |  |  |  |  |  | 1 |  |  |  | 1 |
|  | H13 | .T.............G.. |  |  |  |  |  |  |  |  |  |  | 1 |  |  |  | 1 |
|  | H14 | ..C............... |  |  |  |  |  |  |  |  |  |  | 1 |  |  |  | 1 |
|  | H15 | ....T.......A...T. |  |  |  |  |  |  |  |  |  |  |  |  |  | 1 | 1 |
|  | H16 | ............T...T. |  | 1 |  |  |  |  |  |  |  |  |  |  |  |  | 1 |
|  | H17 | ...C............T. |  |  |  |  |  |  |  | 1 |  |  |  |  |  |  | 1 |
|  | H18 | .T...........C.... |  |  |  | 1 |  |  |  |  |  |  |  |  |  |  | 1 |
|  | H19 | T...T...........T. |  |  |  |  | 1 |  |  |  |  |  |  |  |  |  | 1 |
|  | H20 | ....T....T......T. |  |  |  |  | 1 |  |  |  |  |  |  |  |  |  | 1 |
| Network 2 | H21 | .T....A..G-C...... |  | 16 | 1 |  |  |  |  |  |  |  |  |  |  |  | 17 |
|  | H22 | .T....A.TG-C...... |  | 2 |  |  |  |  |  |  |  |  |  |  |  |  | 2 |
|  | H23 | .T....A..A-C...... |  |  | 1 |  |  |  |  |  |  |  |  |  |  |  | 1 |

BG: Barra de Guaratiba, E1S: Estrela 1S, E2S: Estrela 2S, J1S: Jaiba 1S, J2S: Jaiba 2S, Mar: Marajó, Mes: Mesquita, Pan: Pancas, Ter: Teresina, Jac: Jacobina, Lap: Lapinha, Nat: Natal, S1S: Sobral 1S, S2S: Sobral 2S.
