# Supplementary material for: Molecular and Behavioral Differentiation among Brazilian Populations of Lutzomyia longipalpis (Diptera: Psychodidae: Phlebotominae)
Source: PLoS Negl Trop Dis. 2009 Jan 27;3(1):e365. doi: 10.1371/journal.pntd.0000365 (PMC2628317; doi:10.1371/journal.pntd.0000365)
Supplement: Alternative Language Abstract S1 — Translation of the abstract into Portuguese by Luiz G. S. R. Bauzer (0.01 MB PDF) [file pntd.0000365.s009.pdf]

Translation of the abstract into Portuguese provided by Luiz G. S. R. Bauzer

Resumo

Introdução: *Lutzomyia longipalpis* é o principal vetor da leishmaniose visceral americana. Existem fortes evidências de que *L. longipalpis* é um complexo de espécies, mas até recentemente a existência de espécies irmãs entre populações brasileiras era considerado um assunto controverso. Além disso, ainda não há um consenso quanto ao número de espécies que ocorrem neste complexo.

Metodologia/Principais Resultados: Usando *period*, um gene que controla os ritmos circadianos e que afeta a periodicidade nos intervalos entre pulsos do som de corte de machos de *Drosophila melanogaster* e espécies próximas, nós analisamos o polimorfismo molecular em um número de amostras de *L. longipalpis* de diferentes regiões do Brasil e comparamos os resultados com nossos dados previamente publicados usando o mesmo marcador. Estudamos também os sons de cópula e os feromônios dos machos de algumas destas populações. Os resultados obtidos sugerem a existência de dois grupos principais de populações no Brasil, um grupo representando uma única espécie do complexo, com machos produzindo sons de cópula do tipo Burst e o feromônio cembreno-1; e um segundo grupo, mais heterogêneo, que provavelmente representa um número de espécies incipientes, produzindo diferentes combinações de sons do tipo Pulsado e feromônios.

Conclusões/Importância: Os resultados revelam um elevado nível de complexidade na divergência e no fluxo gênico entre populações brasileiras do complexo de espécies *L. longipalpis.* Isto levanta importantes questões acerca das conseqüências epidemiológicas deste processo incipiente de especiação.
