# Supplementary material for: Molecular and Behavioral Differentiation among Brazilian Populations of Lutzomyia longipalpis (Diptera: Psychodidae: Phlebotominae)
Source: PLoS Negl Trop Dis. 2009 Jan 27;3(1):e365. doi: 10.1371/journal.pntd.0000365 (PMC2628317; doi:10.1371/journal.pntd.0000365)
Supplement: Alternative Language Abstract S2 — Translation of the abstract into Spanish by Alejandra Saori Araki (0.01 MB PDF) [file pntd.0000365.s010.pdf]

Translation of the abstract into Spanish provided by Alejandra Saori Araki

Resumen

Introducción: *Lutzomyia longipalpis* es el principal vector de la leishmaniasis visceral Americana. Existen evidencias sólidas de que *L. longipalpis* es un complejo de especies; sin embargo, hasta hace poco la existencia de especies hermanas en Brasil era considerada controvertida. Actualmente no existe un consenso general sobre el número de especies que conforman este complejo.

Metodología/Principales Resultados: Para analizar el polimorfismo molecular y la divergencia genética en un número de muestras de *L. longipalpis* provenientes de diferentes regiones del Brasil, fue utilizado el gen *period*, el cual controla aspectos de los ritmos circadianos y la periodicidad de los intervalos entre pulsos del sonido de cortejo de *Drosophila melanogaster* y de especies próximas. Los resultados obtenidos fueron comparados con los datos de este mismo marcador molecular previamente publicados. También fueron analizados los sonidos de las cópulas y las feromonas producidas por algunas de estas poblaciones. Los resultados sugieren la existencia de dos principales grupos de poblaciones en Brasil. El primer grupo es representado por los machos de una única especie que producen el sonido de cópula del tipo Burst y la feromona cembreno-1. El segundo grupo, el cual es más heterogéneo y que probablemente representa un número de especies incipientes, produce diferentes combinaciones de feromonas y de sonidos del tipo Pulsado.

Conclusiones/Importancia: Los resultados muestran un elevado nivel de complejidad tanto de la divergencia como del flujo génico entre las poblaciones brasileras del complejo de especies *L. longipalpis*. Esto plantea importantes cuestiones relacionadas a las consecuencias epidemiológicas del proceso incipiente de especiación.
